# Coordinated Expression of Two Types of Low-Threshold K^+^ Channels Establishes Unique Single Spiking of Mauthner Cells among Segmentally Homologous Neurons in the Zebrafish Hindbrain

**DOI:** 10.1523/ENEURO.0249-17.2017

**Published:** 2017-10-23

**Authors:** Takaki Watanabe, Takashi Shimazaki, Yoichi Oda

**Affiliations:** Division of Biological Science, Graduate School of Science, Nagoya University, Nagoya, 464-8602, Japan

**Keywords:** Kv1.1, Kv7.4, Kvβ2, Mauthner cell, phasic firing, simulation

## Abstract

Expression of different ion channels permits homologously-generated neurons to acquire different types of excitability and thus code various kinds of input information. Mauthner (M) series neurons in the teleost hindbrain consist of M cells and their morphological homologs, which are repeated in adjacent segments and share auditory inputs. When excited, M cells generate a single spike at the onset of abrupt stimuli, while their homologs encode input intensity with firing frequency. Our previous study in zebrafish showed that immature M cells burst phasically at 2 d postfertilization (dpf) and acquire single spiking at 4 dpf by specific expression of auxiliary Kvβ2 subunits in M cells in association with common expression of Kv1.1 channels in the M series. Here, we further reveal the ionic mechanisms underlying this functional differentiation. Pharmacological blocking of Kv7/KCNQ in addition to Kv1 altered mature M cells to fire tonically, similar to the homologs. In contrast, blocking either channel alone caused M cells to burst phasically. M cells at 2 dpf fired tonically after blocking Kv7. *In situ* hybridization revealed specific Kv7.4/KCNQ4 expression in M cells at 2 dpf. Kv7.4 and Kv1.1 channels expressed in *Xenopus* oocytes exhibited low-threshold outward currents with slow and fast rise times, while coexpression of Kvβ2 accelerated and increased Kv1.1 currents, respectively. Computational models, modified from a mouse cochlear neuron model, demonstrated that Kv7.4 channels suppress repetitive firing to produce spike-frequency adaptation, while Kvβ2-associated Kv1.1 channels increase firing threshold and decrease the onset latency of spiking. Altogether, coordinated expression of these low-threshold K^+^ channels with Kvβ2 functionally differentiates M cells among homologous neurons.

## Significance Statement

Sensory information is often processed in neural populations with different levels of excitability that extract distinct features of stimuli. It is known that mutations in the low-threshold K^+^ channels, Kv7.4 and Kv1.1, lead to deafness or ataxia in humans, respectively, and losing either channel results in sensory processing impairments due to disrupted phasic excitability in mice. However, how these channels differentially contribute to phasic excitability in a sensory system remains unclear. Here, we show that Kv7.4 and Kv1.1 channels with Kvβ2 subunits have distinct roles in phasic-firing in zebrafish hindbrain neurons. Coordinated expression of these channels is developmentally regulated to ensure that among segmentally homologous, tonically bursting neurons, specific neurons acquire single spiking.

## Introduction

Neurons exhibit varied intrinsic excitability, as represented by temporal firing patterns in response to step depolarization. These are basically classified as either phasic- or tonic-firing patterns by the respective presence or absence of spike-frequency adaptation (Carr and Soares, 2002; Prescott and De Koninck, 2002; Maravall et al., 2004; Kalluri et al., 2010). Heterogeneity in firing threshold, duration, onset latency, and spike-frequency adaptation are also observed among phasic-firing neurons, even in specific neural populations (Locke and Nerbonne, 1997; Prescott and De Koninck, 2002; Soares et al., 2002; Reid et al., 2004). Although intrinsic firing properties are acquired developmentally from immature firing to the phasic or tonic type (Nakamura and Takahashi, 2007; Iwasaki et al., 2008; Hoffpauir et al., 2010; Marrs and Spirou, 2012), the underlying molecular mechanisms remain unclear.

Zebrafish and goldfish Mauthner (M) cells, a pair of giant reticulospinal neurons (RSNs) located in the fourth segment of the hindbrain, are known as a vertebrate model that generates only a single action potential (AP) at the onset of noxious sensory input, thereby triggering fast escape behavior (Zottoli, 1977; Nakayama and Oda, 2004; Kohashi and Oda, 2008). In contrast, two paired RSNs that are repeated in adjacent hindbrain segments (MiD2cm and MiD3cm) are morphologically homologous to M cells, and fire tonically with regular spiking at frequencies dependent on input intensity (Nakayama and Oda, 2004). Because M cells and M-cell homologs, collectively called the “M series” (Lee and Eaton, 1991), share direct projections from auditory afferents (Nakayama and Oda, 2004; Szabo et al., 2007), they are thought to play a role in initiating escape by encoding the onset and intensity of sound, respectively (Oertel, 1999). Because the M series is thought to be evolutionarily duplicated (Metcalfe et al., 1986), we hypothesized that functional differentiation of intrinsic excitability arises from segmental duplication of a common ancestral neuron accompanied by subsequent variation. Our previous zebrafish study indicated that single spiking of M cells is developmentally acquired at 4 d postfertilization (dpf) from immature phasic-firing by enhancing surface expression of voltage-gated K^+^ (Kv) channel α-subunits, *Kv1.1a/kcna1a* (also known as zKv1.1a), via later developmental expression of auxiliary Kvβ2 subunits *Kvβ2b/kcnab2b* (also known as zKvβ2b; Watanabe et al., 2014). However, the phasic-firing pattern of 4-dpf M cells after pharmacologically blocking Kv1.1 channels substantially differs from repetitive firing at 2 dpf. These results raise the possibility that other ion channels contribute developmentally to distinct phasic-firing properties specific to M cells among the M series.

Here, we sought to determine the ionic basis for developmentally-acquired differentiation of firing properties among this neural population. We focused on low-threshold K^+^ channels, including the Kv1 and Kv7/KCNQ families (Johnston et al., 2010), which are activated around the resting membrane potential and play a role in generating phasic-firing in sympathetic, auditory, and vestibular mammalian neurons (Brew and Forsythe, 1995; Wang and McKinnon, 1995; Kalluri et al., 2010). Although they have a similar low-threshold activation voltage, Kv7/KCNQ channels exhibit slow activation-kinetics (Wang et al., 1998), whereas Kv1 channels show rapid activation (Hopkins et al., 1994). However, the differential contributions of these channels to phasic firing remains poorly understood.

In this study, we performed *in vivo* whole-cell recordings from M-series neurons in zebrafish embryos and larvae, and examined the effects of pharmacological manipulations of low-threshold K^+^ channels on firing patterns. We first demonstrate that Kv7/KCNQ is responsible for producing phasic behavior in M cells from 2 dpf, which is distinct from the later contribution of Kv1.1. Second, expression analysis of Kv7 α-subunits, electrophysiological assessment of *Xenopus* oocytes, and computational modeling revealed that early expression of Kv7.4/KCNQ4 in M cells mediates slowly-activating currents to produce phasic bursting. Third, later expression of Kvβ2 enhances inhibition of repetitive firing by Kv1.1, resulting in single spiking. Together, the contributions of two different low-threshold Kv channels with an auxiliary subunit differentiate unique M-cell phasic-firing properties from a common tonic-firing property conserved among the M series.

## Materials and Methods

### Animals

Zebrafish (*Danio rerio*) wild-type and transgenic strains, *Tol-026* (RRID:ZFIN_ZDB-GENO-120508-16) and *Tol-056* (RRID:ZFIN_ZDB-GENO-120316-108), expressing green fluorescent protein (GFP) in M cells and the M-cell homologs MiD2cm and MiD3cm (Kohashi et al., 2012; Watanabe et al., 2014) were reared and staged using established standard protocol. All procedures were performed in compliance with the guidelines approved by the Animal Care and Use Committee of Nagoya University (approval numbers 14-5 and 15-4).

### *In vivo* whole-cell recording

*Tol-026* or *Tol-056* strain embryos and larvae at 56–64 h postfertilization (hpf; 2 dpf) and 102–181 hpf (4–7 dpf) were processed as described previously (Watanabe et al., 2014). In brief, to allow access to M cells or M-cell homologs, embryos or larvae were anesthetized with 0.02% tricaine mesylate (MS-222, Sigma-Aldrich), immobilized with 1 mM D-tubocurarine (Sigma-Aldrich), and then pinned and operated on in a silicon dish filled with extracellular solution containing: 134 mM NaCl, 2.9 mM KCl, 1.2 mM MgCl_2_, 2.1 mM CaCl_2_, 10 mM HEPES, and 10 mM glucose, adjusted to pH 7.8 with NaOH. Whole-cell recordings from M cells and their homologs were obtained using a MultiClamp 700B amplifier (Molecular Devices) and digitizer (Digidata 1440A; Molecular Devices) at a sampling rate of 50 kHz. Patch-clamp electrodes for whole-cell recordings were pulled from borosilicate glass (GD-1.5; Narishige) and filled with intracellular solution containing: 119 mM K-gluconate, 6 mM KCl, 2 mM MgCl_2_, 10 mM HEPES, 10 mM EGTA, and 4 mM Na_2_ATP (at 290 mOsm and pH 7.2), and 0.005% Alexa Fluor 594 hydrazide (Life Technologies). Electrode resistance ranged from 3–8 MΩ. During current-clamp recordings, 6,7-dinitroquinoxaline-2,3-dione (DNQX; Tocris Bioscience) and strychnine (Sigma-Aldrich) were added to the extracellular solution at a final concentration of 50 and 5 μM, respectively. For pharmacological experiments, 10,10-bis(4-pyridinylmethyl)-9(10H)-anthracenone (XE991; Tocris Bioscience) and dendrotoxin-I (DTX; Peptide Institute) were added to the bath at a final concentration of 10 μM and 100 nM, respectively. Data were acquired 20 min after adding the pharmacological solutions, and cells with resting membrane potentials lower than −70 mV were analyzed using Clampfit 10 software (Molecular Devices, RRID:SCR_011323). The liquid junction potential (15 mV) was calculated and corrected for data from M cells or their homologs.

### Cloning of Kv7/KCNQ channels

Zebrafish Kv7/KCNQ channels comprising Kv7.1/KCNQ1, Kv7.2/KCNQ2, Kv7.3/KCNQ3, Kv7.4/KCNQ4, and Kv7.5/KCNQ5 were identified by a blast search of a zebrafish genomic database (Zv9) using protein sequences from their mammalian counterparts. The N and C termini of a single *Kv7.4*/*kcnq4* gene were separately located in adjacent genomic regions of zebrafish ENSDARG00000089490 and ENSDARG00000089559, respectively. We performed a polymerase chain reaction following reverse transcription (RT-PCR) with total RNA extracted from embryos (2 dpf) using the following primers: *Kv7.1a*/*kcnq1a*, 5′-GCTCGAGAGTATTCCTCATTGTGCTTTCCTGC, 5′-GTAGCCAATAGTGGTTACTGTTACC; *Kv7.1b*/*kcnq1b*, 5′-GCTCGAGGTGCTTCCTGTATCACTTCCTC, 5′-CTGCCGATGGATAAAAACCACTG; *Kv7.2a*/*kcnq2a*, 5′-GCTCGAGATGGTGAAGAAATCCGCCAACG, 5′-GAGAAACCTCAGACTCCTTATGG; *Kv7.2b*/*kcnq2b*, 5′-GCTCGAGATCACTCTGACCACCATCGG, 5′-GCACTTTAGATGGACTGTCTTCAATG; *Kv7.3*/*kcnq3*, 5′-GCTCGAGGTTTTGGGATGTCTGATTCTGTCG, 5′-CGTCTGCATAGGTGTCAAAGTC; *Kv7.4*/*kcnq4*, 5′-GCTCGAGATGCTGGGTAGTCCTTCCAATAAC, 5′-GGAATTCTCACTCCATGTTACCGCTGTC; *Kv7.5a*/*kcnq5a*, 5′-GCTCGAGAGTCACGCACAACGACTGAAG, 5′-GAACGAGACACTCCACTACC; *Kv7.5b*/*kcnq5b*, 5′-GCTCGAGGAAGGTGAGGCTAACGTTACTG, 5′-GGAGTTGTTCTGGTTGAGGTTC. Truncated or full-length cDNA segments were cloned in a pCR4Blunt-TOPO vector (Life Technologies). After sequencing, full-length amino acid sequences from Zv9 were aligned across vertebrates using GENETYX software (GENETYX), enabling the construction of phylogenetic trees (Clustal W method).

### *In situ* hybridization

We performed whole-mount and on-section *in situ* hybridization using wild-type embryos (2 dpf) and larvae (5 dpf) with immunostaining and retrograde labeling of RSNs, respectively, as described previously (Watanabe et al., 2014). Briefly, digoxigenin (DIG)-labeled antisense RNA probes, using Kv7 genes as targets and Kv1.1a and Kvβ2b as positive controls, were synthesized *in vitro* using truncated or full-length cDNA, followed by alkaline hydrolyzis at 70°C for 10 min to generate 0.5–1.0 kb probes. At 2 dpf, pigmentation-prevented embryos (obtained after 24 hpf by 0.003% 1-phenyl-2-thiourea (PTU) incubation for 24–30 h; Karlsson et al., 2001) were anesthetized with 0.02% MS-222 until immobilized and then fixed at 48–54 hpf in 4% (w/v) paraformaldehyde (PFA) in PBS. Fixed embryos were treated sequentially with 25%, 50%, 75%, and 100% methanol/PBS 0.1% Tween 20 for 5 min each time, and stored with 100% methanol for over 2 h at −20°C. After proteinase K treatment (10 μg/ml; Roche) for 40–60 min, embryos were refixed and hybridized with RNA probes at 65°C for 12–16 h. After washing at 65°C, embryos were incubated with horseradish peroxidase-conjugated anti-DIG antibody (1:2000; Roche) and monoclonal 3A10 antibody [1:50; Developmental Studies Hybridoma Bank (DSHB), RRID:AB_531874] for 12–16 h at 4°C. RNA probes were detected using a tyramide signal amplification (Cardin et al., 2009) kit with Alexa Fluor 488 (Life Technologies) and an anti-mouse IgG secondary antibody conjugated with Alexa Fluor 568 (1:2000; Roche). After removing the yolk, dorsal images of whole-mounted embryos were obtained by confocal microscopy (FV300; Olympus). For 5 dpf, wild-type larvae were injected with 10% (w/v) Alexa Fluor 568 fixable dextran 10,000 MW (Life Technologies) into the caudal spinal cord at 4 dpf, and then fixed in 4% PFA/PBS at 120–125 hpf. After methanol treatment, larvae were incubated in 30% sucrose and embedded in Tissue-Teq O.C.T. compound (Sakura). Thick horizontal sections of larvae (20-μm) were obtained using a cryostat (CM1850; Leica), and treated with proteinase K (5 μg/ml) for 15 min, followed by refixation for 20 min. Sections were then processed using the same protocol as for whole-mount *in situ* hybridization.

### Two-electrode voltage-clamp recordings in *Xenopus* oocytes

*Xenopus laevis* oocytes were collected and treated as previously described (Watanabe et al., 2014), and injected using a Drummond microinjector with 46 nl cRNA (250 ng/μl) synthesized from full-length zebrafish *Kv7.4*/*kcnq4*, *Kv1.1a*/*kcna1a*, and *Kvβ2b*/*kcnab2b* cDNA using a mMESSAGE mMACHINE Kit (Life Technologies). The volume ratio of *Kv1.1a* and *Kvβ2b* cRNA solution was 1:5. K^+^ currents were recorded using two electrodes with a resistance of 0.5–1.0 MΩ (when filled with 3 M KCl), and an Axoclamp 2B amplifier (Molecular Devices). During recordings, oocytes were perfused in a chamber with a bath solution containing: 96 mM NaMeSO_4_, 2 mM KCl, 1 mM MgCl_2_, 1 mM CaCl_2_, and 5 mM HEPES, adjusted to pH 7.6 with NaOH. Niflumic acid (0.3 mM) was added to block endogenous chloride currents (Hopkins et al., 1994). Transient capacitance and linear leakage currents were subtracted using a P/4 procedure. The conductance-voltage plots were fitted by a standard Boltzmann equation (*G* = *G*max/[1 + exp(*V*_1/2_ − *V*)/*k*], where V_1/2_ is the voltage of half-maximal activation, and *k* is the slope factor). Data were analyzed and fitted using Clampfit 10 software (Molecular Devices).

### Computational model

We simulated low-threshold K^+^ currents and M cell firing in a single-compartment model of a *Xenopus* oocyte and M cell, respectively, using NEURON simulator (version 7.3, RRID:SCR_005393; Hines and Carnevale, 2001). Our model channel generator and firing simulator are publicly available on Model DB repository (https://senselab.med.yale.edu/ModelDB; accession # 232813) and as Extended Data 1. The simulations were based on a Hodgkin-Huxley (H-H) model of a mammalian ventral cochlear nucleus neuron, which like an M cell, possesses a single-spiking property (Rothman and Manis, 2003b). We constructed single cylindrical compartments for the *Xenopus* oocyte model (20-μm length and 20-μm diameter) and M-cell model (40-μm length and 40-μm diameter), possessing 12 pF and 50 pF, respectively (specific membrane capacitance: c_m_ = 1 μF/cm^2^). These compartments enabled us to directly observe the effects of ion conductances on the cell without cable properties derived from an axon and dendrites or non-uniform localization of ion channels. The parameters of the H-H model for low-threshold K^+^ conductance of each zebrafish Kv7.4 (*g*_Kv7.4_), Kv1.1 (*g*_Kv1.1_), and Kv1.1 with Kvβ2 (*g_Kv1.1+Kvβ2_*) were fitted to the data from the *Xenopus* oocyte system described above, by simulating voltage-clamp recordings at a virtual 20°C. The model M cell included the following maximal conductances: 6000 ns, voltage-gated Na^+^ conductance (*ḡ*_Nav_: 120 ms/cm^2^); 1500 ns, high-threshold K^+^ conductance (*ḡ*_high_: 30 ms/cm^2^); 300 ns, A-type K^+^ conductance (*ḡ*_A_: 6 ms/cm^2^); and 12 ns, leak conductance (*ḡ*_leak_: 0.24 ms/cm^2^) and arbitrary low-threshold K^+^ conductance (*ḡ*_Kv7.4_, *ḡ*_Kv1.1_, *ḡ_Kv1.1+Kvβ2_*). Additionally, H-H models of *g*_Nav_, *g*_high_, and *g*_A_ were modified from the study by Rothman and Manis (Rothman and Manis, 2003b) to fit M-cell firing and AP wave form. The reversal potentials of Na^+^, K^+^, and the leakage conductances were set to +30 mV, −90 mV, and −85 mV, respectively, and the resting potential of the M-cell model was set to a default of −85 mV, as observed *in vivo* (Watanabe et al., 2014). Current-clamp simulation was conducted at a virtual temperature of 25°C, and the threshold current (1T) was found by 1 pA increments via current injection. The combination effects of Kv7.4 and Kv1.1 for 2-dpf M cells, or Kv7.4 and Kv1.1 with Kvβ2 for 4-dpf M cells were simulated by implementation of (*ḡ*_Kv7.4_ and *ḡ*_Kv1.1_) or (*ḡ*_Kv7.4_ and *ḡ_Kv1.1+Kvβ2_*). Overall, 1071 firing patterns (21 × 51 combinations) were produced by increments of 100 ns for *ḡ*_Kv7.4_ and 20 ns for *ḡ*_Kv1.1_ or *ḡ_Kv1.1+Kvβ2_*, ranging from 0 to 2000 ns (40 ms/cm^2^) and from 0 to 1000 ns (20 ms/cm^2^), respectively. Firing properties were analyzed using MATLAB R2013a software (MathWorks, RRID:SCR_001622).

10.1523/ENEURO.0249-17.2017.ed1 Extended Data 1 The model channel generator and firing simulator on NEURON simulator. The NEURON model files encode the channel generator and firing simulator for simulating development and differentiation of the M-cell excitability. The channel generator enables us to generate arbitrary Na^+^ and K^+^ channels by changing parameters of a Hodgkin-Huxley model under emulation of two-electrode voltage-clamp recordings in *Xenopus* oocyte system. The firing simulator simulates current-clamp recordings to generate firing pattern of the model M cell, which are implemented with arbitrary-generated Na^+^ and K^+^ conductances and low-threshold K^+^ channels Kv7.4/KCNQ4 and sole Kv1.1 or Kv1.1 coexpressed with Kvβ2. Download Extended data, ZIP file.

### Statistical analysis

The pharmacological experiments in the M cells were analyzed using Mann–Whitney *U* tests for two independent data sets. Two-way repeated measures ANOVA was used for interspike interval (ISI) comparisons because ISI was repeatedly measured from the same cell at different time points from ISI_1st_ to ISI_12th_. We used the Kruskal-Wallis test with the *post hoc* Bonferroni-corrected Mann–Whitney *U* test for analysis of the *Xenopus* oocyte system. Statistical analysis was conducted in EZR (Kanda, 2013). All data are represented as mean ± SEM.

## Results

### Coordinated contribution of Kv7/KCNQ and Kv1 channels to single spiking in zebrafish M cells

**Figure 1.**
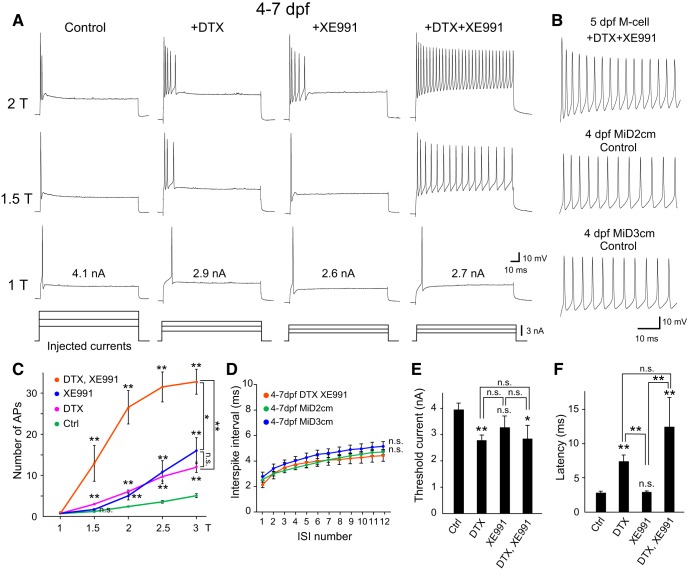
Effect of DTX and/or XE991 on single spiking in developed M cells. ***A***, Firing response of M cells at 4–7 dpf [upper three rows of traces elicited by step depolarizing currents (lower traces) of 1×, 1.5×, and 2× threshold intensity (T), as represented in nA on 1T traces] before and after bath application of either 100 nM DTX, 10 μM XE991, or both. ***B***, Initial phase of repetitive firing in response to 2T current injection recorded from M cells with DTX and XE991 treatment, and untreated (control) MiD2cm and MiD3cm cells at 4 dpf. ***C***, Number of APs elicited during a depolarizing current pulse (100 ms) plotted against current intensity (T). The number of APs significantly increased after combined DTX and XE991 treatment compared with each separately. ***D***, Summary data for the ISI of repetitive firing in response to 2T current injection against the interval number. After combined DTX and XE991 treatment, M cells at 4–7 dpf exhibited ISIs that were similar to untreated MiD2cm and MiD3cm cells at the same developmental stage. ***E***, ***F***, The effect of DTX and/or XE991 on threshold current (***E***) and onset latency of the first spike at 1T (***F***). Treatment of DTX, but not XE991, reduced the threshold current and increased spiking latency of M cells. n.s., not significant. *p < 0.05, **p < 0.01, Mann–Whitney *U* test (control, *n* = 14; +DTX, *n* = 8; +XE991, *n* = 7; +DTX + XE991, *n* = 6). Two-way repeated measures ANOVA was used for ISI (MiD2cm, *n* = 8; MiD3cm, *n* = 7).

To identify the ion channels responsible for establishing the unique single-spiking property of M cells among M-series neurons, we first examined the effect of pharmacologically blocking low-threshold K^+^ channels on firing patterns in response to step depolarization. *In vivo* whole-cell recordings from zebrafish M cells at 4-7 dpf revealed typical single spiking at the onset of step depolarization, which contrasts the tonic firing of the M-cell homologs repeated in adjacent segments (i.e., MiD2cm and MiD3cm; Fig. 1). Bathing with either DTX or XE991, specific blockers for Kv1 or Kv7 channels, respectively, caused M cells to burst phasically (Fig. 1*A*). After treatment with both DTX and XE991, M cells showed tonic firing or regular spiking at frequencies that depended on the injected current intensity, similar to the segmental homologs (Fig. 1*A*,*B*; Nakayama and Oda, 2004; Watanabe et al., 2014). We quantitatively illustrated the effect of K^+^ channel blockers by plotting the number of spikes elicited by depolarizing current pulses against the current intensity (Fig. 1*C*). We found no significant difference in the ISI compared with M-cell homologs (Fig. 1*D*). These results demonstrate that both DTX- and XE991-sensitive Kv1 and Kv7, respectively, are responsible for acquisition of the single-spiking property by M cells. Hence, coordinated expression of both types of low-threshold K^+^ channels is likely a key step for functional differentiation of M cells as possessing a single-spiking activity pattern that distinguishes them from their homologous neurons.

It should be noted that DTX- and XE991-sensitive K^+^ channels may contribute differentially to M-cell excitability, because DTX treatment decreased threshold current intensity for spiking, and prolonged the time to spike initiation (spike latency) from stimulus onset at threshold intensity, whereas XE991 treatment alone did not affect these properties (Fig. 1*E*,*F*). Moreover, there was a tendency for a more prolonged spike latency with both DTX and XE991 treatment (Fig. 1*F*; see Discussion).

### Kv7/KCNQ channels are required for phasic bursting of immature M cells

**Figure 2.**
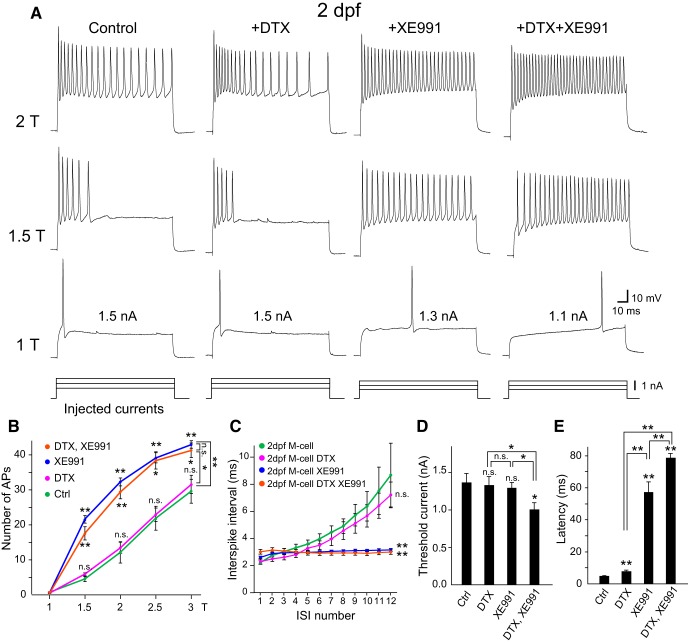
Effect of DTX and/or XE991 on phasic bursting of immature M cells. ***A***, Firing response from M cells at 2 dpf. XE991 treatment caused phasic bursting M cells (control) to fire tonically (+XE991, +DTX + XE991), whereas DTX treatment did not (except for a slight delay of spike onset). ***B***, Summary graph of the blocker effect at 2 dpf showing that the number of APs elicited during 100-ms current pulses significantly increased after XE991 treatment. ***C***, In controls, M cells at 2 dpf showed spike accommodation with a gradual increase in ISI, whereas regular spiking with constant ISI was observed after XE991 treatment. Note that DTX application had no additional effect. ***D***, The threshold current was significantly reduced after combined DTX and XE991 treatment. ***E***, Spike latency greatly increased after XE991 treatment. n.s., not significant. *p < 0.05, **p < 0.01, Mann–Whitney *U* test (control, *n* = 6; +DTX, *n* = 6; +XE991, *n* = 10; +DTX + XE991, *n* = 7). Two-way repeated measures ANOVA was used for ISI.

Since immature M cells are known to burst (Fig. 2; Watanabe et al., 2014), we next examined the contribution of both types of low-threshold K^+^ channels on M-cell firing during early development. Blocking Kv1 channels with DTX treatment had little effect on immature zebrafish M cells at 2 dpf (Fig. 2*A*), as we previously reported (Watanabe et al., 2014). In contrast, blocking Kv7 channels with XE991 treatment significantly enhanced excitability, especially at the later phase of step depolarization, resulting in tonic firing at regular intervals (Fig. 2*A*). This quality was also exhibited by M-cell homologs in control preparations. We summarized the effects by plotting spike number against depolarizing current intensity (Fig. 2*B*). Immature M cells exhibited spike adaptation with increasing ISI at the later phase, with adaptation completely abolished by XE991 (Fig. 2*A*,*C*). XE991 treatment also affected the spike latency of M cells (Fig. 2*E*). These results indicate that XE991-sensitive K^+^ channels mainly contribute to phasic bursting of M cells at the early stage of development. However, some contribution of DTX-sensitive channels cannot be excluded because threshold current and spike latency were additionally affected by the combined application of DTX and XE991 (Fig. 2*D*,*E*).

Overall, these results suggest that Kv7 channels are already expressed in 2-dpf M cells, and that they are required for the establishment of phasic bursting with strong spike-frequency adaptation. Further, this raises the possibility that Kv7 channels are specifically expressed in M cells among M-series neurons.

### Kv7.4/KCNQ4 channels are specifically expressed in M cells among M-series neurons

**Figure 3.**
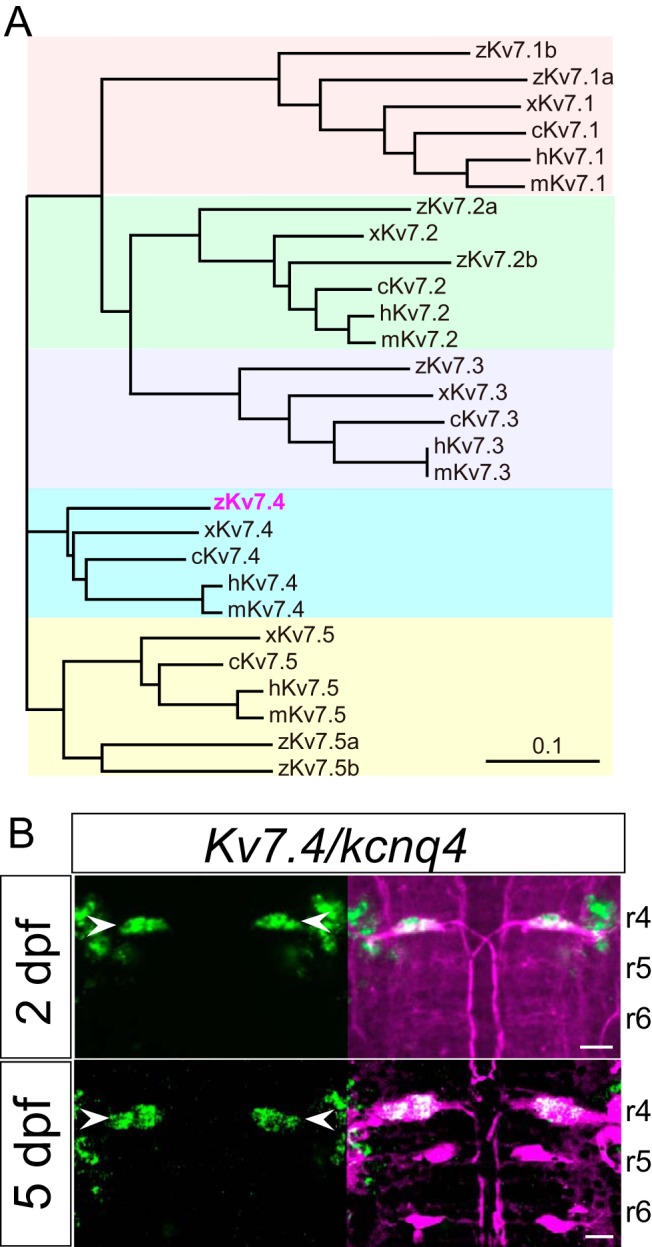
Expression of *Kv7.4/Kcnq4* mRNA in M cells during development. ***A***, Phylogenetic tree of α-subunit proteins of XE991-sensitive voltage-gated K^+^ channel Kv7/KCNQ family members in zebrafish, *Xenopus*, chick, mouse, and human (z, x, c, m, and h as the gene prefix, respectively), and showing identification of zebrafish Kv7.4 (magenta). ***B***, Dorsal views of rhombomeres 4, 5, and 6 (r4, r5, and r6) in the hindbrain at 2 dpf (top) and 5 dpf (bottom) after *in situ* hybridization using a *Kv7.4* antisense probe (green). *Kv7.4* mRNA was expressed in M cells (arrowhead) at 2 and 5 dpf, but not in MiD2cm and MiD3cm cells, labeled (magenta) immunohistochemically (with 3A10 antibody, at 2 dpf) or retrogradely (at 5 dpf). Scale bar, 20 μm.

To test this possibility, we investigated the expression of Kv7 channels in M-series neurons during development. In zebrafish, Kv7/KCNQ α-subunit genes consist of *Kv7.1a*, *Kv7.1b*, *Kv7.2a*, *Kv7.2b*, *Kv7.3*, *Kv7.4*, *Kv7.5a*, and *Kv7.5b* (Fig. 3*A*). Zebrafish *Kv7.4* has appeared to consist of two genes in different genomic regions (Wu et al., 2014). However, due to incomplete genomic sequencing, we were only able to amplify a single full-length *Kv7.4*/*kcnq4* cDNA segment by RT-PCR from these two genomic regions, in which N- and C-terminal *Kv7.4*/*kcnq4* are separately located (see Materials and Methods). *In situ* hybridization in 2- and 5-dpf zebrafish revealed that among these genes, only *Kv7.4/kcnq4* mRNA was specifically expressed in M cells, but this expression did not take place in the MiD2cm or MiD3cm (Fig. 3*B*). In addition, *Kv7.4/kcnq4* mRNA was also expressed in some vestibular neurons located in the lateral M-cell region (Fig. 3*B*). Other Kv7 subunits were not detected in M-series neurons (data not shown), demonstrating Kv7.4 probe specificity. These results suggest that homomeric Kv7.4 channels are already specifically formed in M cells at 2 dpf, in contrast to Kv1.1 α-subunits (*kcna1a/Kv1.1a*), which appear in all M-series neurons but exhibit enhanced expression via Kvβ2 (*kcnab2b/Kvβ2b*) auxiliary subunits after 2 dpf (Watanabe et al., 2014).

### Zebrafish Kv7.4 channels form low-threshold K^+^ currents with slow activation

**Figure 4.**
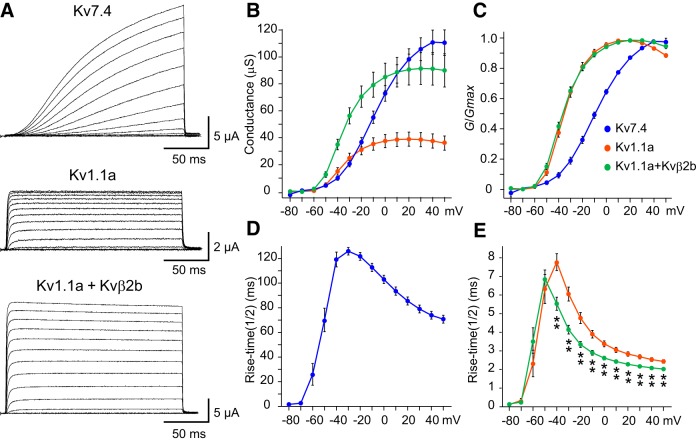
Gating properties of zebrafish Kv7.4 and Kv1.1 with/without Kvβ2 in *Xenopus* oocytes. ***A***, Voltage-gated outward currents recorded from *Xenopus* oocytes expressing zebrafish Kv7.4 (top), Kv1.1a alone (middle), or Kv1.1a with Kvβ2b subunit (bottom). Command voltages (V) ranging from −80 to +50 mV in 10 mV steps were applied for 200 ms. ***B***, Conductance (*G*, μs) at peak current amplitude was plotted as a function of the command voltage. ***C***, The *G*-*V* relationship normalized to maximum value (*G*_max_) shows that currents elicited above −60 mV had different open probabilities. ***D***, ***E***, Rise time to half-maximum activation (t_1/2_) of Kv7.4 (***D***) above −60 mV was significantly different from Kv1.1a with and without Kvβ2b (***E***; *p* < 0.01). **p < 0.01, Kruskal-Wallis test with *post hoc* Bonferroni correction (Kv7.4, *n* = 20; Kv1.1a, *n* = 32; Kv1.1a + Kvβ2b, *n* = 26).

As demonstrated, Kv7.4 and Kv1.1 channels appear to contribute differentially to the control of M-cell firing. To assess the electrophysiological kinetic differences between the zebrafish Kv7.4 and Kv1.1, we compared voltage-gated currents in *Xenopus* oocytes expressing either of these channels. Zebrafish Kv7.4 channels expressed in oocytes showed slowly-activating outward currents at command voltages above −60 mV (Fig. 4*A*). The threshold voltage was comparable to that of Kv1.1 (Fig. 4*B*,*C*; *G*/*G*_max_ at −60 mV, *p* = 0.357, Mann–Whitney *U* test; Kv7.4, *n* = 20; Kv1.1a, *n* = 32). However, the rise time of Kv7.4 channel activation was several ten-fold slower than that of Kv1.1 channels (Fig. 4*D*,*E*). Interestingly, when coexpressed with auxiliary Kvβ2 subunits, Kv1.1 channel activation was significantly accelerated, with a rise time that was ∼2 ms faster above −40 mV (Fig. 4*E*) and an increased current amplitude (Fig. 4*B*; Watanabe et al., 2014). That these channels have different kinetics is also demonstrated by the half activation voltage (V_1/2_) and slope *k* parameters of the Boltzmann equation (Table 1). Therefore, the expression of slowly-activating Kv7.4 channels in M cells appears to contribute to strong spike-frequency adaptation in phasic bursting at 2 dpf, and the expression of fast-activating Kv1.1 channels associated with Kvβ2 subunits establishes single spiking after 3 dpf.

**Table 1.** Comparison of the parameters of the Boltzmann equation in *Xenopus* oocytes system is shown

	*n*	*V*_1/2_ (mV)	*k*
Kv7.4	20	−10.1 ± 1.7	15.4 ± 0.7
Kv1.1a	32	−35.4 ± 1.4**	7.4 ± 0.3**
Kv1.1a + Kvβ2b	26	−36.6 ± 1.1**	9.4 ± 0.5**

All values are represented as mean ± SEM. **p < 0.01, Mann–Whitney *U* tests against the value of Kv7.4.

### Coordinated expression of Kv7.4 and Kv1.1 associated with Kvβ2 underlies developmental differentiation of M-cell excitability among homologous neurons

**Figure 5.**
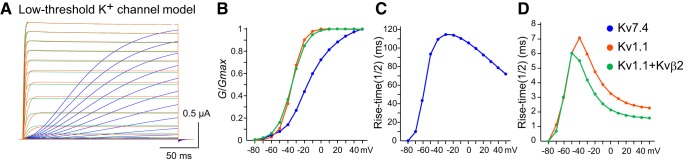
Computational model of low-threshold K^+^ channels. ***A***, Voltage-gated outward currents of model zebrafish Kv7.4 (blue), Kv1.1 (orange), and Kv1.1 channels coexpressed with Kvβ2 (green) represented by a Hodgkin-Huxley equation. ***B***–***D***, Normalized *G*-*V* plots (***B***), and rise time (t_1/2_) of model Kv7.4 (***C***), Kv1.1 and Kv1.1 + Kvβ2 (***D***) resemble those obtained in *Xenopus* oocytes (Fig. 4).

**Figure 6.**
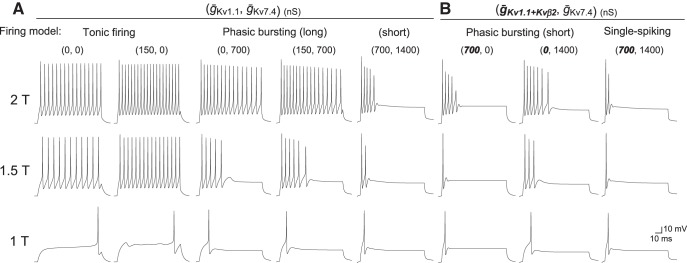
M-cell firing model integrating low-threshold K^+^ channels in response to long step currents. ***A***, ***B***, Representative current-clamp simulation of a model neuron integrating two types of low-threshold K^+^ channels associated with Kvβ2 subunits into a basic tonic-firing model. Long step currents of 1T, 1.5T, and 2T were injected. Maximum conductances (*ḡ*_Kv1.1_, *ḡ*_Kv7.4_; ***A***) or *(*ḡ*_Kv1.1+Kvβ2_, *ḡ**_Kv7.4_; ***B***) are indicated in parentheses. Bold italic numbers represent conductances associated with Kvβ2. Expression of low-threshold K^+^ channels alters the tonic-firing model to exhibit phasic bursting with long or short firing duration and single spiking, as observed with *in vivo* M-cell recordings (Figs. 1, 2).

Next, we examined the role of coexpression of these two low-threshold K^+^ channels and Kvβ2 subunits on developmental acquisition of M-cell excitability by examining firing behavior in model cells. We focused on temporal patterns of firing in response to depolarization as well as the latency and threshold of firing. We represented the zebrafish Kv7.4 and Kv1.1 channels with and without Kvβ2 subunits via a basic H-H equation (Fig. 5) and integrated them into a control regular-spiking model (Fig. 6). Voltage-dependent open probability and time-dependent kinetics of the channels were reproduced in the model (Fig. 5*B*-*D*, respectively) as observed in the *Xenopus* oocyte system (Fig. 4). It is nearly impossible to precisely determine all of the ionic conductances in M cells via voltage-clamp recording due to large leak currents through gap junctions in huge dendrites (Pereda et al., 2013; Watanabe et al., 2014). Thus, the control model cell, which reproduces regular spiking as shown by MiD2/3cm (Fig. 1*B*; Watanabe et al., 2014) or M cells after both DTX and XE991 treatment (Figs. 1, 2), is based on that of mouse cochlear neurons (Rothman and Manis, 2003b). Mouse cochlear neurons express voltage-gated Na^+^ channels, high-threshold K^+^ channels, and A-type K^+^ channels, and fire tonically in response to step depolarization [Fig. 6*A*; tonic firing (*ḡ*_Kv1.1_, *ḡ*_Kv7.4_: 0, 0)]. The model cells that additionally expressed some degree of Kv1.1 conductance still exhibited tonic firing but at slightly higher frequencies [Fig. 6*A*; (*ḡ*_Kv1.1_, *ḡ*_Kv7.4_: 150, 0)], as observed in 2-dpf M cells after XE991 treatment. Integration of both Kv1.1 and Kv7.4 conductance (*ḡ*_Kv1.1_, *ḡ*_Kv7.4_: 150, 700) and Kv7.4 conductance only (*ḡ*_Kv1.1_, *ḡ*_Kv7.4_: 0, 700) reproduced phasic bursting of M cells at 2 dpf and those after DTX treatment, respectively [Fig. 6*A*; phasic bursting (long)]. The spike latency increased when the Kv1.1 conductance was subtracted from the 2-dpf M-cell model, as observed after DTX treatment. To test the effect of Kvβ2 subunit coexpression on firing, we increased the model Kv1.1 conductance along with an acceleration in activation kinetics of ∼2 ms above −40 mV (*ḡ_Kv1.1+Kvβ2_*: 700), as observed in oocytes (Figs. 5*D*, 6*B* ). The model cell with either enhanced Kv1.1 conductance with Kvβ2 or twice as many Kv7.4 channels showed phasic bursting [Fig. 6*B*; phasic bursting (short) (*ḡ_Kv1.1+Kvβ2_*, *ḡ*_Kv7.4_: 700, 0 or 0, 1400)]. The model cell exhibited single spiking only when both conductances were integrated [Fig. 6*B*; single spiking (*ḡ_Kv1.1+Kvβ2_*, *ḡ*_Kv7.4_: 700, 1400)]. The model cell (*ḡ*_Kv1.1_, *ḡ*_Kv7.4_: 700, 1400) expressing the two channels but without acceleration by Kvβ2 still exhibited short phasic bursting activated by a lower threshold current [Fig. 6*A*; phasic bursting (short)]. These simulations demonstrate that the coordinated expression of Kv7.4 and Kv1.1 coexpressed with Kvβ2 accounts for the developmental change in M-cell excitability from phasic long bursting at 2 dpf to single spiking at 4 dpf. Moreover, Kv7.4 conductance appears to increase during M-cell development (see Discussion).

**Figure 7.**
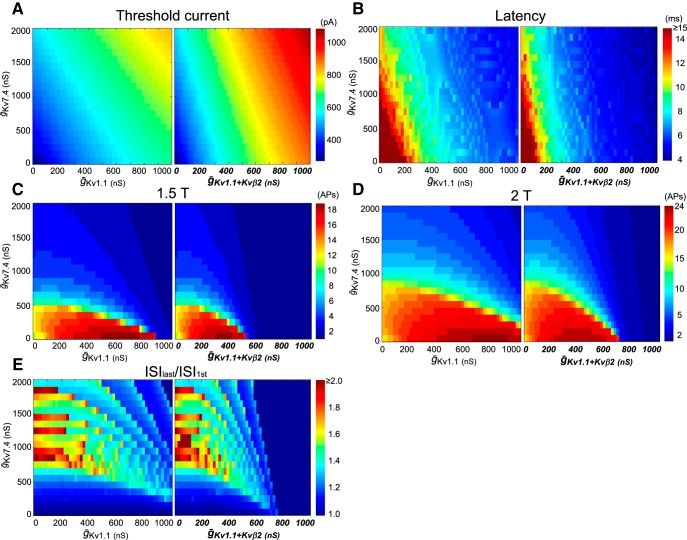
Comprehensive analysis of firing parameters in the M-cell model. ***A–E***, Firing parameters of low-threshold K^+^ channel-integrated model cells with different combinations of (*ḡ*_Kv1.1_, *ḡ*_Kv7.4_; left) or (*ḡ_Kv1.1+Kvβ2_*, *ḡ*_Kv7.4_; right), with respect to threshold current (T; ***A***), onset latency of first AP at 1T (***B***), number of elicited APs during 100 ms at 1.5T (***C***) and 2T (***D***), and ISI_last_/ISI_1st_ at 2T (***E***). In each panel, 1071 firing patterns (21 × 51 combinations) were produced by increments of 100 ns for *ḡ*_Kv7.4_ and 20 ns for *ḡ*_Kv1.1_ or *ḡ_Kv1.1+Kvβ2_*, ranging from 0-2000 ns (40 ms/cm^2^) and 0-1000 ns (20 ms/cm^2^), respectively. The Kv1.1 model (*ḡ*_Kv1.1_, *ḡ*_Kv7.4_: 480, 0) and Kv1.1 + Kvβ2 model (*ḡ_Kv1.1+Kvβ2_*, *ḡ_Kv7.4_*: 240, 0) had a similar threshold current (around 500 pA; ***A***). The Kv1.1 model (*ḡ*_Kv1.1_, *ḡ*_Kv7.4_: 920, 0) showed two spikes at 1.5T, and this firing rate was also seen in the Kv1.1 + Kvβ2 model (*ḡ_Kv1.1+Kvβ2_*, *ḡ*_Kv7.4_: 540, 0; ***C***), indicating that faster activation of Kv1.1 by Kvβ2 efficiently suppressed repetitive firing.

In addition to the typical examples above, we comprehensively analyzed the relationship between the amount of low-threshold K^+^ conductance and model cell excitability, as shown in Figure 7. While recruitment of Kv1.1 conductance in tonic-firing cells increased the threshold current and shortened the firing onset latency, especially when coexpressed with Kvβ2, the addition of Kv7.4 conductance had a relatively small effect (Fig. 7*A*,*B*). Increasing Kv7.4 conductance resulted in a gradual suppression of bursting (Fig. 7*C*,*D*) accompanied by strong spike-frequency adaptation (Fig. 7*E*; ordinate), while increasing Kv1.1 conductance had a relatively small effect (Fig. 7*E*; abscissa). Thus, Kv7.4 and Kv1.1 channels play distinct firing property roles, while Kvβ2 subunits enhance the effect of Kv1.1 channels. It should be noted that Kvβ2 coexpression had a significant impact on phasic firing, even at smaller conductances.

**Figure 8.**
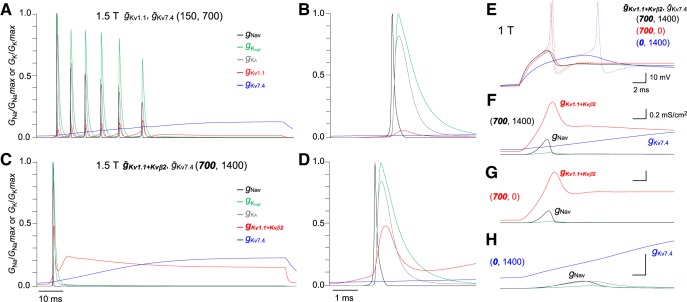
Na^+^ and K^+^ conductance mechanisms for producing characteristic phasic properties. ***A–D***, Representative dynamics of voltage-gated Na^+^ and K^+^ channel conductance for *g*_Nav_ (black), *g*_high_ (green), *g*_A_ (gray), *g*_Kv7.4_ (blue), and *g*_Kv1.1_ or *g_Kv1.1+Kvβ2_* (red) during current-clamp stimulation. These conductances were normalized to maximum values of Na^+^ or among K^+^ conductance at 1.5T using a 2-dpf (***A***, ***B***) and 4-dpf (***C***, ***D***) M-cell model, shown in Figure 7*A*,*B*, respectively. Conductance dynamics at the beginning of current injection in ***B***, ***D*** show the first AP generation of ***A***, ***C***, respectively. ***E–H***, Changes in membrane potential at subthreshold (solid line, below 1*–*2 pA of 1T), and 1T (dashed line) in the 4-dpf M-cell model (black), XE991-treated model (red), and DTX-treated model (blue; ***E***). The corresponding Na^+^ and K^+^ conductance in each model during subthreshold firing (***F*–*H***). Na^+^ conductance became much larger after fast activation of K^+^ conductance (*ḡ_Kv1.1+Kvβ2_*; ***F***, ***G***) compared with slow activation of K^+^ conductance (*ḡ*_Kv7.4_; ***H***).

We represented behaviors of different types of voltage-gated channels during phasic firing of M cells using the model cell (Fig. 8). During phasic bursting, Kv1.1 channels are periodically activated from the onset of current injection, whereas activation of Kv7.4 channels is gradually increased at the later phase of repetitive firing (Fig. 8*A*,*B*; red vs blue). Thus, fast activation of Kv1.1 channels with low density presumably increases firing frequency rather than suppressing later firing, whereas slow activation of Kv7.4 channels mainly affects spike-frequency adaptation. In the case of single spiking, activation of Kv1.1 with Kvβ2 rose rapidly immediately at onset, and maintained high levels until the end of spiking. In contrast, Kv7.4 activation increased gradually for phasic bursting (Fig. 8*C*,*D*). These data suggest that the fast activation and high conductance of Kv1.1 channels coexpressed with Kvβ2 strongly inhibits bursting after the first AP. Further, at juxta-threshold voltages, Kv1.1 with Kvβ2 also contributed to fast activation of voltage-gated Na^+^ conductance, whereas Kv7.4 did not (Fig. 8*E*–*H*). The differing contribution of Kv1.1 channels with Kvβ2 and Kv7.4 channels on Na^+^ channels may underlie the differences in spike latency of M cells during DTX and XE991 treatment.

**Figure 9.**
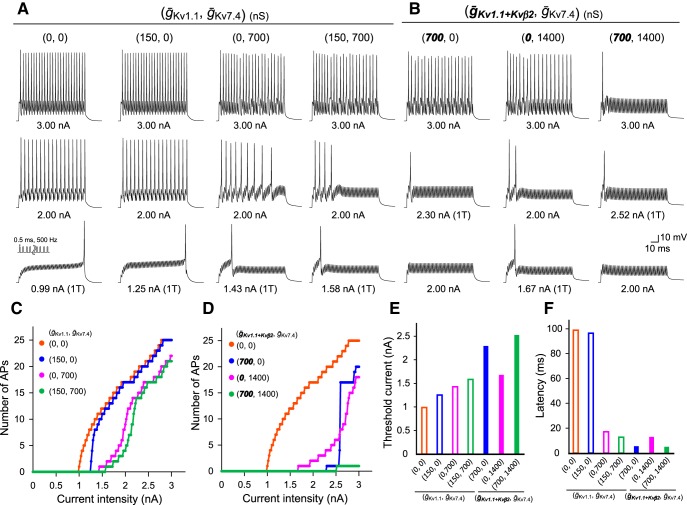
Simulation of M-cell firing in response to repetitive short-pulse currents. ***A***, ***B***, Current-clamp simulation of each model neuron (*ḡ*_Kv1.1_, *ḡ*_Kv7.4_; ***A***) or (*ḡ_Kv1.1+Kvβ2_*, *ḡ*_Kv7.4_; ***B***), which was the same as in Figure 6 except that repetitive short-pulse currents (0.5 ms) of 1T, 2 nA, and 3 nA with 500 Hz were given for 100 ms. ***C***, ***D***, Number of APs simulated in (*ḡ*_Kv1.1_, *ḡ*_Kv7.4_; ***C***) or (*ḡ_Kv1.1+Kvβ2_*, *ḡ*_Kv7.4_; ***D***) are plotted during the current injection in 20-pA increments up to 3 nA. ***E***, ***F***, Threshold current (***E***) and onset latency of the first spike at 1T (***F***) for each model neuron (*ḡ*_Kv1.1_, *ḡ*_Kv7.4_) or (*ḡ_Kv1.1+Kvβ2_*, *ḡ*_Kv7.4_).

Finally, we estimated a role for the expression of the two K^+^ channels on M cells in the firing response to auditory inputs by applying repetitive short-pulse depolarizing currents (0.5 ms, 0–3 nA) at 500 Hz (Fig. 9). M-series neurons receive direct auditory afferent nerves that burst at 100-1000 Hz in goldfish (Fay, 1995). Firing responses of the model (*ḡ*_Kv1.1_, *ḡ*_Kv7.4_) or (*ḡ_Kv1.1+Kvβ2_*, *ḡ*_Kv7.4_) were quite similar to those evoked by step current injection (Figs. 6, 9*A*,*B* ). A tonic firing model [*ḡ*_Kv1.1_, *ḡ*_Kv7.4_: 0, 0 or 150, 0] quickly increased firing frequency just above the threshold currents, whereas a phasic bursting model [*ḡ*_Kv1.1_, *ḡ*_Kv7.4_:0, 700 or 150, 700] gradually increased the number of APs with a lower frequency in a range of relatively small intensities above the threshold (Fig. 9*A*,*C*). A single-spiking model [*ḡ_Kv1.1+Kvβ2_*, *ḡ*_Kv7.4_: 700, 1400] generated a single AP in response to large current intensities (2.5-3 nA), while a short phasic-bursting model [*ḡ_Kv1.1+Kvβ2_*, *ḡ*_Kv7.4_: 700, 0 or 0, 1400] produced repetitive firing with a high frequency in response to the same large current intensities (Fig. 9*B*,*D*). The effects of integration of these channels on threshold current and latency of the first AP had the same tendency for both M cells *in vivo* (Fig. 1, 2) and those in the simulation with step current injection (Figs. 6, 9*E*,*F*).

Taken together, these results demonstrate that coordinated expression of slowly-activating Kv7.4 channels, fast-activating Kv1.1 channels, and enhancement by auxiliary Kvβ2 subunits produces heterogeneity of phasic firing in M cells with different developmental stages, thus further differentiating these neurons from homologous counterparts.

## Discussion

Using the zebrafish as a model to understand how unique single spiking is produced based on a tonic-firing property, we have identified ionic mechanisms underlying developmental differentiation of M-cell excitability among segmentally homologous hindbrain neurons. Single-spiking mature M cells begin to fire tonically, as observed in the M-cell homologs, when two types of low-threshold K^+^ currents are blocked (Fig. 1), indicating that the ionic basis for tonic firing is conserved in the M series. Conversely, these low-threshold K^+^ currents are responsible for differentiation of M-cell excitability from that of the M-cell homologs. In contrast to common Kv1.1 channel expression among the M series (Watanabe et al., 2014), slowly-activating Kv7.4 channels and auxiliary Kvβ2 subunits that accelerate and increase Kv1.1 currents are expressed specifically in M cells from embryonic and later developmental stages, respectively. The expression of Kv7.4 channels and Kvβ2 associated-Kv1.1 channels during development plays different roles in phasic firing such that the combination of these activities induces M cells to acquire their characteristic single-spiking pattern.

Other mechanisms are known to generate phasic or single spiking properties. A change in intrinsic membrane properties from tonic to phasic firing has been well investigated in spiny lobster culture preparations of stomatogastric ganglion neurons, which can be accounted for by increased Ca^2+^ currents and decreased K^+^ currents (Turrigiano et al., 1994; Turrigiano et al., 1995). In rat ventral horn neurons, pharmacological blocking of persistent Na^+^ currents causes tonic-firing neurons to fire a single spike (Theiss et al., 2007). Simulation analysis of RSNs in frog tadpoles suggests that single spiking evoked by step current injection is not a cellular property but the consequence of a shunting effect resulting from electrical coupling via gap-junction connections among a neural population (Hull et al., 2015). Because we did not test the contribution of these currents to M-cell firing, we cannot exclude the possibility that Ca^2+^ currents, persistent Na^+^ currents, and gap-junction connections might underlie M-cell firing properties. However, even if they exist in M cells, their contribution will presumably only have a minimal effect on generating single spiking of M cells because of the necessity and sufficiency of coexpression of the two low-threshold K^+^ channels for single spiking.

### Coexpression of Kv7.4 and Kv1.1 channels in vertebrate sensory neurons

Which neurons express both Kv7 and Kv1 channels, like M cells, in vertebrates? In zebrafish, vestibular nucleus neurons express Kv7.4 and Kv1.1 channels from 2 dpf, and Kvβ2 subunits from 5 dpf (Fig. 3*B*; Watanabe et al., 2014). In rat vestibular ganglion neurons both Kv7 and Kv1 channels are required for single-spiking vestibular afferent neurons (Kalluri et al., 2010), which express K^+^ channels including Kv7.4 and/or Kv1.1 (Hurley et al., 2006; Lysakowski et al., 2011). Interestingly, phasic spiral ganglion neurons located at the base of the cochlea exhibit stronger expression of Kv7.4 and/or Kv1.1 than tonic neurons located at the apex (Beisel et al., 2000; Adamson et al., 2002). Furthermore, coexpression of Kv7 and Kv1 channels associated with Kvβ subunits has been indirectly suggested not only in vestibular and auditory neurons but also phasic trigeminal ganglia neurons and dorsal root ganglia neurons, as well as their secondary sensory neurons, in mice and rats (Rhodes et al., 1996; Kharkovets et al., 2000; Rasband et al., 2001; Caminos et al., 2005; Heidenreich et al., 2012). Thus, coordinated expression of two types of low-threshold K^+^ channels is observed in the vertebrate sensory system.

Kv7.4 gene mutations in humans and mice lead to impairments of hearing and touch sensation caused by dysfunction and progressive degeneration of outer hair cells (Kharkovets et al., 2006) or disruption of phasic neurons in trigeminal and dorsal root ganglia (Heidenreich et al., 2012). Kv1.1 mutations in humans and knock-out in mice are known to cause episodic ataxia type 1 associated with epilepsy, which results from dysfunction of the cerebellum, hippocampus, cortex, and peripheral nervous system (Smart et al., 1998; D'Adamo et al., 2015). In the auditory system of Kv1.1 knock-out mice, phasic firing of the ventral cochlear nucleus and medial nucleus of trapezoid body neurons impairs temporal spiking precision and increases firing frequency (Brew et al., 2003; Kopp-Scheinpflug et al., 2003; Gittelman and Tempel, 2006), causing reduced performance of interaural intensity difference in the lateral superior olive (Karcz et al., 2011). These findings demonstrate that losing either of two low-threshold K^+^ channels in sensory neurons results in sensory dysfunction by altering phasic neurons to display hyperexcitability or loss of temporal precision. Therefore, coordinated expression in these neurons appears to play a crucial role in detecting phasic or onset timing and raising the firing threshold against sensory stimuli, as likely in the case of M cells. To reveal the physiologic role of these channels in M cell-initiated escape behavior, further behavioral experiments combined with M cell-specific gene manipulation of these channels is needed.

### Distinct roles of Kv7.4 and Kv1.1 channels in phasic-firing properties

Previous computational model studies have reconstructed phasic-firing patterns by implementing either Kv1 channels (Rothman and Manis, 2003b; Catacuzzeno et al., 2008) or Kv7 channels (Zaika et al., 2006) in tonic-firing cells. Here, as the cochlear nucleus neuron model (Rothman and Manis, 2003a), our model also indicated that the suppression of repetitive firing by Kv1.1 channels alone requires large conductance (Fig. 7*C*,*D*).

Slowly-activating Kv7.4 channels work primarily via spike-frequency adaptation to produce shorter bursts at frequencies that increase with injected currents (Figs. 6, 7). This role for Kv7/KCNQ channels in spike-frequency adaptation has been emphasized in rat sympathetic neurons and mouse CA1 pyramidal neurons, in which heteromeric channels composed of Kv7.2/KCNQ2 and Kv7.3/KCNQ3 subunits are expressed (Wang et al., 1998; Chung et al., 2006; Soh et al., 2014). Phasic-bursting neurons in the mouse cerebral cortex also exhibit firing with frequencies proportional to input intensity and longer onset spike latency, i.e., over 10 ms (Soh et al., 2014). Thus, neurons expressing only Kv7 channels in addition to conventional voltage-gated Na^+^ and high-threshold K^+^ channels burst phasically with spike-frequency adaptation, as is the case with M cells at 2 dpf.

Here, for the first time, we have shown the necessity of integrated expression of Kv7.4 channels and Kv1.1 channels with Kvβ2 auxiliary subunits to produce single spiking as the most extreme pattern of phasic firing. Further, fast outward currents through the Kv1.1 channels substantially increased the threshold current for spiking, as reported before in rat vestibular neurons (Kalluri et al., 2010). Kv1.1 channels associated with Kvβ2 shortened the spike latency (Figs. 6, 7, 9). Therefore, neurons expressing Kv1 channels associated with Kvβ2 encode onset-timing information in response to relatively large inputs, but still burst at an input intensity-dependent frequency [Figs. 6*B*, *9B* ; phasic busting (short)]. Together, neurons expressing both Kv7 and Kvβ2-associated Kv1 channels extract precise onset-timing information without duration and intensity, as is the case with M cells and rat vestibular neurons (Kohashi and Oda, 2008; Kalluri et al., 2010).

The effect of combined DTX and XE991 treatment was greater than either treatment at 2 and 4 dpf in M cells (Figs. 1*F*, 2*E*), and this phenomenon was replicated in model cells (Figs. 7*B*, 8*E*–*H*, 9*F* ). Thus, in addition to Kv1, Kv7 currents also shorten spike latency but the effect is much smaller than Kv1 currents. This difference may be mainly due to the distinct impact on Na^+^ conductance activation (Fig. 8*E*–*H*). Faster activation of Na^+^ conductance may be attributed to reduced membrane resistance and a shorter time constant of the depolarizing phase before spiking, which is caused by rapidly increasing K^+^ currents from Kv1.1 channels associated with Kvβ2 rather than slowly-activating Kv7.4 channels. Conversely, without low-threshold K^+^ currents, the membrane depolarization time constant becomes much longer (as passive membranes demonstrate), and then a slower depolarization only weakly activates Na^+^ conductance, resulting in a considerably longer spike latency of tonic firing.

### Developmental increases in Kv7.4 and Kv1.1 conductance

Our comprehensive simulation produced pairs of conductances for low-threshold K^+^ channels that best explain our experimental data, with a relatively higher conductance for Kv7.4 versus Kv1.1 (Fig. 6). The higher Kv7.4 conductance presumably originated from incomplete modification of basic H-H models of *g*_Nav_, *g*_high_, and *g*_A_ based on cochlear neurons (Rothman and Manis, 2003b) along with the differences in voltage range and rise time for activation of Kv7.4 versus Kv1.1 channels (Fig. 4). Our modifications of *g*_Nav_, *g*_high_, and *g*_A_ were arbitrarily adjusted by fitting model firing and AP wave form to experimental data of 4-dpf M cells, which somehow affected our model results throughout development. A comparison between the zebrafish Kv7.4 and the other Kv7 channels in the mouse indicated that zebrafish Kv7.4 channels have more depolarized *V*_1/2_ parameters compared with mouse KCNQ1, KCNQ2 and KCNQ4 channels (Nakajo and Kubo, 2005). This suggests that these channels play different roles in zebrafish M cells and mouse neurons.

Our M-cell model indicated that increasing the Kv7.4 and Kv1.1 currents by 2 and 4.7 times, respectively, sufficiently reproduced M-cell development and data from previous pharmacological studies. Increases in Kv1.1 currents can be explained by enhanced surface expression associated with Kvβ2, as observed in the *Xenopus* oocyte system (Fig. 5), and/or possibly by developmental accumulation of gene expression in M cells. Indeed, this increase in gene expression corresponds to the development of auditory and vestibular neurons in mammals and birds, and these neurons can alter intrinsic firing properties in response to increases in Kv1 currents and expression levels (Gamkrelidze et al., 1998; Nakamura and Takahashi, 2007; Iwasaki et al., 2008; Iwasaki et al., 2012). Alternatively, the molecular basis for the developmental increases in Kv7.4 conductance may not simply be explained by an increase in gene expression, because strong expression was already observed at 2 dpf (Fig. 3*B*).

What possible mechanisms underlie the increase of Kv7.4 currents? Alternative splicing variants in mouse Kv7.4/KCNQ4 (variants 1–4) exhibit distinct current amplitudes when expressed in Chinese hamster ovarian cells (Xu et al., 2007). These isoforms are differentially expressed in inner hair cells, afferent spiral ganglion neurons (Beisel et al., 2005), and vestibular ganglion neurons (Rocha-Sanchez et al., 2007), suggesting nervous system regulation of conductance by Kv7.4 isoforms. Moreover, mammalian Kv7 conductance is modulated and inhibited by phosphorylation of C-terminal sites via protein kinase C (PKC; Hoshi et al., 2003; Nakajo and Kubo, 2005). Further, membrane surface expression and kinetics are regulated by association with potassium voltage-gated channel subfamily E (KCNE) β-subunits (KCNE1–5; Strutz-Seebohm et al., 2006; Pongs and Schwarz, 2010). In this study, we found that the zebrafish Kv7.4 is comparable to a short-type variant of the mouse homolog, with conserved PKC-phosphorylated residues in the C terminus, shown by amino acid sequence alignment across vertebrates (Fig. 3*A*; Nakajo and Kubo, 2005). M cells express PKCγ protein, at least from 2 dpf (Patten et al., 2007). Zebrafish KCNE1 and KCNE4 subunits are present in the zebrafish genome database (Zv9), but not all homologs have been identified. Further studies are required to examine possible changes in splicing isoform expression, and to determine if regulation of Kv7.4 channels via PKC or KCNE β-subunits is involved in M-cell excitability during development.

In conclusion, M-series neurons may represent the simplest model for explaining the developmental establishment of different phasic-firing properties from a tonic-firing property in a neural population via coordinated expression of low-threshold K^+^ channel subunits. Because Kv1, Kvβ, and Kv7 gene families are widely expressed in the vertebrate nervous system, it is likely that distinct activation-kinetics and expression or conductance levels of these low-threshold K^+^ channels play important roles in producing heterogeneity of phasic-firing properties.

## Synthesis

Reviewing Editor: Muriel Thoby-Brisson, CNRS UMR 5287 Université Bordeaux 2 Decisions are customarily a result of the Reviewing Editor and the peer reviewers coming together and discussing their recommendations until a consensus is reached. When revisions are invited, a fact-based synthesis statement explaining their decision and outlining what is needed to prepare a revision will be listed below. The following reviewer(s) agreed to reveal their identity: Mark Masino, Wen-Chang Li

Dear Authors,

The two reviewers and I agreed on the potential interest of your study in which the combination between pharmacological tests and computer modelling provides two lines of evidence that converge nicely to support the conclusion that the coordinated expression of these K+ channels is important in the maturation of M-cell single-spiking properties. However we also all found that the study deserves to be placed in a much broader context. You definitively need to cite more widely to explain that you are addressing a problem interesting to a wider readership.

Specific comments:

This study provides systematic and convincing evidences that the unique single-spiking property of the Mauthner cells (part of the M-series of segmentally homologous hindbrain neurons) is developmentally mediated by a combination of expression of two low-threshold potassium channels. The main goal of the work was to identify the ionic mechanism responsible for the different firing properties among the M-series neurons. The authors focused on low-threshold potassium channels because they have been shown to play a role in phasic-firing patterns in other vertebrate neurons. First, they showed that the developmentally regulated expression of a potassium channel with slow activation kinetics (Kv7/KCNQ) generates the phasic-firing in Mauthner cells, which was supported by expression analysis in Xenopus oocytes and computer simulations. Finally, later expression of an auxiliary potassium channel subunit (KvB2) was shown to enhance Kv1.1 and thereby inhibited repetitive firing. However despite how convincing and detailed this work is, the authors fail to develop a compelling argument for the significance of the results in a broader context.

Major Comments:

- A major weakness of this manuscript is the need for broader significance. For instance: Lines 59-68, why is this work important beyond developmental regulation of differential firing properties? It is necessary for the authors to relate the results of this work back into their proposal that differential expression of ion channels produce different levels of excitability among various members of a neuronal population and that this permits the extraction of distinct features of sensory stimuli.

Also in the discussion, Lines 441-44: The authors fail to develop a compelling argument for the significance of the results in a broader context. They approach it when suggesting that the coordinated expression of these potassium channels is related to the phasic or onset of sensory stimuli (lines 61 and 441-44), however, the logic is not well developed and, so, is reaches well beyond the scope of what is reasonable to interpret based on the results presented.

- It has been elegantly explored using modelling of STG neurons by Eve Marder's lab that different combinations of ionic currents can give rise to seemingly very similar firing behaviour. Without most ionic currents revealed in M-cells using voltage-clamp recordings or pharmacology, the conclusion should be drawn with more caution. For single firing properties, other currents like persistent sodium currents (Theiss et al., 2007) or gap-junctional connections (Hull et al., 2015) in reticulospinal or spinal neurons can make contributions, too. The discussion should be expanded to cover some other possibilities that can give rise to similar single firing behaviour.

- Another weakness that may not be resolved in this study is the use of mouse cochlear neuron as their control model cell because of the difficulties in analysing the ionic currents in M-cells using voltage-clamp recordings. This is acceptable but should be acknowledged clearly in the abstract and proper discussion should be given to cover if this could affect the conclusion.

- Lines 273-76: The authors did not sufficiently address the interesting observation that spike latency was exacerbated by the combination of DTX and XE991. This effect was greater that additive, so this suggests another mechanism is at play. An alternative is that there is some synergism between the drugs. The authors need to address this result more thoroughly.

- In regards to Rigor and Reproducibility, the authors failed to explain or present the controls administered for the ISH experiments. As it is presented, one cannot confidently state that the expression pattern observed in Figure 3B is specific to the probe.

Minor Comments:

- Lines 183-84: Please provide sufficient information or citations related to the “pigmentation-prevented embryos”. Was this via PTU? If so, provide details of concentration and timing (when applied and for how long).

- Line 184: There is not sufficient information provided regarding the application of paraformaldehyde. Were the embryos/larvae first anesthetized or euthanized prior to fixation? If so, how?

- Line 185: Provide concentration (presumably 100%) and duration the embryos/larvae were treated with Methanol.

- Lines 327-330: It is very difficult to follow the sentence structure for the comparison of V1/2 indicated in the parentheses.

- Lines 330-33: Based on the difficulty following the statement in Lines 327-30, it is not possible to assess the confidence level of this statement.

- Line 391, some explanation is needed to explain how Kv1.1 and KvB2 contribute to fast activation of Na+ conductance.

- Line 396: Suggest that the authors use the term “approximately” or the symbol “~” in place of the term “about”.

- Line 718-719, match legend with fig panels.

- Fig.5A: threshold, not thrshold.

## Author Response

Manuscript Instructions

- The sex of the species studied is not mentioned in the materials and methods section. Please mention “male”, “female”, “x males and x females”, “of either sex”.

We have included this information in the revised manuscript: "of either sex" (line 124).

Reply to reviewer

Major Comments:

- A major weakness of this manuscript is the need for broader significance. For instance: Lines 59-68, why is this work important beyond developmental regulation of differential firing properties? It is necessary for the authors to relate the results of this work back into their proposal that differential expression of ion channels produce different levels of excitability among various members of a neuronal population and that this permits the extraction of distinct features of sensory stimuli.

We appreciate the reviewer's comment regarding the need for a broader significance. Accordingly, we have revised the significance statement (lines 61-68) and described Kv7.4 and Kv1.1 mutations that are observed in human disorders as well as physiological results obtained in the respective knockout mice.

Also in the discussion, Lines 441-44: The authors fail to develop a compelling argument for the significance of the results in a broader context. They approach it when suggesting that the coordinated expression of these potassium channels is related to the phasic or onset of sensory stimuli (lines 61 and 441-448), however, the logic is not well developed and, so, is reaches well beyond the scope of what is reasonable to interpret based on the results presented.

We have not directly shown a physiological role for these channels in M-cell-initiated escape behavior. Hence, further behavioral experiments combined with M-cell-specific gene manipulation of these channels is needed. Instead, we have explained our rationale for the relationship between coordinated Kv7.4 and Kv1.1 expression and physiological significance by citing published papers describing the physiological consequence of these mutations in humans and knockout mice. These papers demonstrate that loss of either of the two low-threshold K+ channels in sensory neurons results in sensory dysfunction. Thus, coordinated expression in these neurons appears to play a crucial role in phasic or onset of sensory stimuli. We have included a detailed version of this discussion in the revised manuscript (lines 463-479).

- It has been elegantly explored using modelling of STG neurons by Eve Marder's lab that different combinations of ionic currents can give rise to seemingly very similar firing behaviour. Without most ionic currents revealed in M-cells using voltage-clamp recordings or pharmacology, the conclusion should be drawn with more caution. For single firing properties, other currents like persistent sodium currents (Theiss et al.,

2007) or gap-junctional connections (Hull et al., 2015) in reticulospinal or spinal neurons can make contributions, too. The discussion should be expanded to cover some other possibilities that can give rise to similar single firing behavior.

As suggested by the reviewer, there are reports describing the mechanisms for phasic firing by Ca2+ currents, persistent Na+ currents, and gap-junction connections. Therefore, we cannot exclude the possibility that these currents might underlie M-cell firing properties because we did not test their contribution to M-cell firing. However, by considering the necessity and sufficiency of coexpression of these two low-threshold K+ channels for single spiking, the contributions of these currents appears to provide only a slight effect on single-spiking of M-cells. We have expanded our discussion to cover these possibilities (lines 434-447).

- Another weakness that may not be resolved in this study is the use of mouse cochlear neuron as their control model cell because of the difficulties in analysing the ionic currents in M-cells using voltage-clamp recordings. This is acceptable but should be acknowledged clearly in the abstract and proper discussion should be given to cover if this could affect the conclusion.

Our computational models were based on the mouse cochlear neuron model but with modifications made to the H-H model of gNav, ghigh, and gA conductances. Our modification of gNav, ghigh, and gA was arbitrarily adjusted by fitting model firing and AP waveform to experimental data of 4 dpf M-cells, which somehow affected our model results throughout development due to incomplete modification. We have described this in the revised manuscript (lines 523-528), and clearly mentioned the modification in the Abstract (line 54) to clearly express our acknowledgement.

- Lines 273-76: The authors did not sufficiently address the interesting observation that spike latency was exacerbated by the combination of DTX and XE991. This effect was greater that additive, so this suggests another mechanism is at play. An alternative is that there is some synergism between the drugs. The authors need to address this result more thoroughly.

We have carefully considered this result and described the tendency for more prolonged spike latency with combined DTX and XE991 treatment in the revised manuscript (lines 283-285). The combined effect of DTX and XE991 treatment was observed in 2 and 4 dpf M-cells and also replicated in model cells. Further, in addition to Kv1, Kv7 currents also shorten spike latency but the effect is much smaller than Kv1 currents. Moreover, we have provided an explanation for how these channels differentially shorten spike latency and why the latency is markedly longer without two low-threshold K+ channels (lines 508-518).

- In regards to Rigor and Reproducibility, the authors failed to explain or present the controls administered for the ISH experiments. As it is presented, one cannot confidently state that the expression pattern observed in Figure 3B is specific to the probe.

We used zebrafish Kv1.1a and Kvβ2b probes as positive controls, which we used in our previous study on M-cells (Watanabe et al., 2014). Kv7.4 probe specificity was suggested by data showing no detectable signal for the other Kv7 subunits despite similarity among this family. We have included this explanation in the Material and Methods (line 183) and Results (lines 318-319) sections.

Minor Comments:

- Lines 183-84: Please provide sufficient information or citations related to the “pigmentation-prevented embryos”. Was this via PTU? If so, provide details of concentration and timing (when applied and for how long).

We have provided information on pigmentation via PTU and cited an appropriate reference (lines 185-186).

- Line 184: There is not sufficient information provided regarding the application of paraformaldehyde. Were the embryos/larvae first anesthetized or euthanized prior to fixation? If so, how?

We have provided information regarding paraformaldehyde fixation and anesthetization (lines 187)

- Line 185: Provide concentration (presumably 100%) and duration the embryos/larvae were treated with Methanol.

We have revised the explanation for methanol treatment (lines 188-190).

- Lines 327-330: It is very difficult to follow the sentence structure for the comparison of V1/2 indicated in the parentheses.

- Lines 330-33: Based on the difficulty following the statement in Lines 327-30, it is not possible to assess the confidence level of this statement.

We have summarized our comparison of the Boltzmann equation parameters in Table 1 (line 336 and Table 1).

- Line 391, some explanation is needed to explain how Kv1.1 and KvB2 contribute to fast activation of Na+ conductance.

As noted above, we have included an explanation for fast activation of Na+ conductance in the Discussion of the revised manuscript (lines 508-518).

- Line 396: Suggest that the authors use the term “approximately” or the symbol “~” in place of the term “about”.

We have corrected the description appropriately (lines 402-403).

- Line 718-719, match legend with fig panels.

We have corrected the symbol (line 792).

- Fig.5A: threshold, not thrshold.

We have corrected the spelling of this word in Figure 5A.
